# Intensity Sensitive Modulation Effect of Theta Burst Form of Median Nerve Stimulation on the Monosynaptic Spinal Reflex

**DOI:** 10.1155/2015/704849

**Published:** 2015-03-04

**Authors:** Kuei-Lin Yeh, Po-Yu Fong, Ying-Zu Huang

**Affiliations:** ^1^Chang Gung University College of Medicine, Taoyuan City 333, Taiwan; ^2^Department of Neurology, Chang Gung Memorial Hospital, Taoyuan City 333, Taiwan; ^3^Neuroscience Research Center, Chang Gung Memorial Hospital, Taoyuan City 333, Taiwan

## Abstract

The effects of electrical stimulation of median nerve with a continuous theta burst pattern (EcTBS) on the spinal H-reflex were studied. Different intensities and durations of EcTBS were given to the median nerve to 11 healthy individuals. The amplitude ratio of the H-reflex to maximum M wave (H/M ratio), corticospinal excitability and inhibition measured using motor evoked potentials (MEPs), short-interval intracortical inhibition and facilitation (SICI/ICF), spinal reciprocal inhibition (RI), and postactivation depression (PAD) were measured before and after EcTBS. In result, the H/M ratio was reduced followed by EcTBS at 90% H-reflex threshold, and the effect lasted longer after 1200 pulses than after 600 pulses of EcTBS. In contrast, EcTBS at 110% threshold facilitated the H/M ratio, while at 80% threshold it had no effect. Maximum M wave, MEPs, SICI/ICF, RI, and PAD all remained unchanged after EcTBS. In conclusion, EcTBS produced lasting effects purely on the H-reflex, probably, through effects on postsynaptic plasticity. The effect of EcTBS depends on the intensity and duration of stimulation. EcTBS is beneficial to research on mechanisms of human plasticity. Moreover, its ability to modulate spinal excitability is expected to have therapeutic benefits on neurological disorders involving spinal cord dysfunction.

## 1. Introduction

Plasticity is an intrinsic reaction to adapt to environmental pressures, physiologic changes, and experiences [1]. There are several ways to induce synaptic long-term potentiation (LTP) and long-term depression (LTD), which are believed to form the basis of plasticity. Although synaptic plasticity has been mostly studied in the brain, it is also evident in the spinal cord in animal and human studies using different stimulation protocols [2–4]. Spinal plasticity can be induced by repetitive electric stimulation in the ventral horn of the rat spinal cord in vitro [2] and the spinal stretch reflex can be modified in monkeys by giving a steady moderate extension torque [3]. In addition, some protocols have been developed that change the excitability of the H-reflex, again supporting the existence of plasticity within the human spinal cord [5].

Spinal plasticity is important in the functional recovery of several neurological disorders. For instance, synaptic plasticity is known to occur simultaneously with axonal sprouting and cellular proliferation after spinal cord injuries (SCIs) and may contribute to improve motor function [6–8]. Another study indicated a direct relation between plasticity and the time course of recovery in rats with lateral hemisection injury of the thoracic spinal cord [9].

In contrast, spinal plasticity may also cause neurological disorders. Spasticity, which is one of the most common sequelae of SCIs and strokes, may be the best example. Although spasticity involves complex changes in the spinal cord and muscles [10–15], it is generally accepted that the loss of supraspinal control leads to the development of spasticity with hyperexcitability of the spinal reflex through the mechanism of plasticity within the spinal cord [16, 17]. Hence, we developed a protocol to modulate spinal plasticity in humans with the intention that it may be applied therapeutically in neurological disease (e.g., spasticity).

So far, only a few noninvasive protocols, including transcutaneous electrical nerve stimulation (TENS) [18], patterned sensory stimulation [19], operant conditioning [20], spinal paired associative stimulation (spinal PAS) [5, 21], and spinal transcutaneous direct current stimulation (tsDCS) [22], have been developed for similar purposes. TENS showed inhibitory effects on the stretch reflex in spasticity of cerebral origin for approximately 45 min after the end of TENS [23], and yet some subjects felt uncomfortable and even painful when receiving TENS due to high frequency waves. Patterned sensory stimulation changed reciprocal Ia inhibition of the H-reflex after 30 min of stimulation [19], although the effect on the size of the H-reflex itself was not reported. Operant conditioning protocols for adjusting the size of the H-reflex have been shown to produce beneficial effects on locomotion of patients with SCI [20, 24]. However, not all patients are capable of performing the protocols. Spinal PAS successfully facilitated the H-reflex at spinal level [5]. However the requirement of stimulation over the primary motor cortex or cervicomedullary junction makes spinal PAS unsuitable for patients with a damaged corticospinal tract. Spinal tsDCS has been shown to produce lasting effects on sensory evoked potentials and postactivation depression of the H-reflex [22, 25]. However, the effect of tsDCS on the H-reflex itself or the ratio of maximum H-reflex and maximum M wave (Mmax) has been inconsistent [25, 26]. Moreover, TENS, spinal PAS, and tsDCS require a relatively lengthy stimulation to produce positive effects.

In the present study, we developed a protocol for producing an inhibitory LTD-like effect in the spinal cord. Continuous theta burst stimulation (cTBS) based on repetitive TMS over M1 has shown its efficiency and efficacy in the suppression of cortical excitability through a LTD-like mechanism [27–31]. We therefore chose to adapt the protocol of cTBS to activate median nerve input to spinal cord. Rather than employing magnetic stimulation, cTBS was applied with a conventional electrical stimulator. By tuning the number and intensity of stimuli, we could identify optimal protocols for producing long-lasting modulation effects on the spinal excitability. Moreover, we tested the effect of EcTBS on motor cortical and corticospinal excitability using transcranial magnetic stimulation (TMS) to measure the motor evoked potential (MEP), short-interval intracortical inhibition (SICI), and intracortical facilitation (ICF). In addition, spinal reciprocal inhibition (RI) and postactivation depression (PAD) were measured to confirm the location of the effect.

## 2. Methods

### 2.1. Subjects

Eleven healthy subjects without history of neurological disorders were recruited. Eight of the subjects (2 males, 6 females; 23.5 ± 0.8 years) underwent the complete experiments and 10 subjects participated in the experiment of spinal reciprocal inhibition. One subject, who did not have a consistent H-reflex in the forearm, was excluded. All participants signed their informed consent prior to the participation. The project protocol was approved by the Institutional Review Board of Chang Gung Memorial Hospital, Taiwan.

### 2.2. EMG Recording

Each subject was seated in a comfortable chair. EMGs were performed with 1 cm-diameter Ag/AgCl-plated surface electrodes placed 2 cm apart over the tested muscles in the dominant hand as instructed separated below in each experiment. The EMG of the flexor carpi radialis muscle (FCR, for H-reflex recording and RI), extensor digitorum communis muscle (EDC, for RI), and abductor pollicis brevis muscle (APB, for TMS tests) were recorded. The EMG was amplified and band-pass filtered (3 Hz to 2 kHz) by Digitimer D360 amplifiers (Digitimer Ltd., Welwyn Garden City, Herts, UK). Signals were recorded at a sampling rate of 5 kHz and stored on the computer for later analysis by Signal software (Cambridge Electronic Design Ltd., Cambridge, UK) through a power 1401 data acquisition interface (Cambridge Electronic Design Ltd., Cambridge, UK). If the target muscle was not relaxed (as monitored with an EMG gain of ×5000) during the test, the data would be scrapped and the test redone.

### 2.3. EcTBS

EcTBS was adapted from the theta burst protocol (TBS) of repetitive transcranial magnetic stimulation (rTMS) [30] and consisted of a 3-pulse burst of 50 Hz electric stimulation (1 ms pulse width) given every 200 ms to the median nerve in the antecubital fossa using a constant current stimulator (DS7A, Digitimer, Welwyn, UK). Two protocols of EcTBS were included in the present study: (1) EcTBS600: 600 pulses of stimuli given in a 40-second train of EcTBS and (2) EcTBS1200: 1200 pulses of stimuli given in an 80-second train of EcTBS. The former was tested at the stimulus intensity of 90% and 110% of H-reflex threshold recorded from FCR, while the latter was tested at 80% and 90% of H-reflex threshold.

### 2.4. Experiment 1: The Effect of EcTBS on H-Reflexes

The effects of EcTBS were firstly evaluated with the maximum H-reflex and Mmax. H-reflexes were recorded from FCR by stimulation of the median nerve. Electrical median nerve stimulation was performed through surface electrodes to stimulate the median nerve in the antecubital fossa with the duration of 1 ms. In the baseline condition, the H-reflex recruitment curve was recorded with the stimulus intensities at 90%, 100%, 120%, 140%, 160%, 180%, and 200% of H-reflex threshold, which was defined as the minimum stimulus current to the evoked-potential with an amplitude of 50 *μ*V, in a random order. A total of 6 trials given every 6–8 sec were recorded in each condition. The intensities that produced the 2 maximum H-reflexes were used for testing the effect of EcTBS afterwards. Mmax was also recorded from the same FCR muscle by using supramaximal stimulation. Then EcTBS was applied on the median nerve. Two maximum H-reflexes (6 trials each) and Mmax were recorded again at 0, 15, 30, 45, and 60 min after the end of cTBS EcTBS. The H/M ratio was calculated by dividing the peak-to-peak amplitude of H-reflex by the peak-to-peak amplitude of Mmax. Two H/M ratios were then averaged for analysis.

We started the experiments with EcTBS600 at 90% and 110% H-reflex threshold intensity and discovered a better inhibitory effect at an intensity of 90% (see below for the results). Since we aimed to find an optimal inhibitory protocol, we doubled the stimulus duration to have EcTBS1200 at 90% and 80% H-reflex threshold for the following study. We expected to achieve a better result by doubling the length of stimulation because studies of rTMS usually find that the effect lasts longer after longer periods of stimulation. In addition, 80% H-reflex threshold may have produced better inhibition than 90%, since there was better inhibition at 90% than at 110% threshold using EcTBS600.

### 2.5. Experiment 2: The Effect of EcTBS on Motor Cortical and Corticospinal Circuits

In this session, TMS was used to evaluate the effect of EcTBS on excitatory and inhibitory circuits in the motor cortex. TMS was given using a hand-held figure of eight coils with a mean loop diameter of 70 mm (Magstim Co., Whiteland, Dyfed, UK) connected to a Magstim Bistim2 machine (Magstim Co., UK). The coil was placed tangentially to the scalp over the “motor hot-spot” with the handle pointing backwards. The “motor hot-spot” was marked on the scalp and defined as the location where magnetic stimulation produced the largest MEP in the APB muscle. APB was selected as the target muscle because like FCR it is innervated by the median nerve but has a clearer MEP than FCR. Short-interval intracortical inhibition and intracortical facilitation (SICI/ICF) were assessed in the baseline condition and at 30 min after the EcTBS1200 at 90% H-reflex threshold. SICI/ICF were tested using a paired-pulse technique with a subthreshold conditioning stimulus followed by a suprathreshold test stimulus of TMS at interstimulus intervals (ISI) of 3, 7, and 10 ms [32]. The conditioning stimulus was set at 80% of the active motor threshold (AMT) and the test stimulus was set at the intensity required to produce an MEP of 1 mV. AMT was defined as the minimum single pulse intensity required to produce an MEP of greater than 200 *μ*V in more than five out of ten trials from the contralateral APB while the subject was maintaining a voluntary contraction of about 20% of the maximum using visual feedback. Subjects received the stimulation in a random order either of the test stimulus alone or of conditioning-test stimuli for a total of 12 trials per condition. The intertrial interval was 4.5–5.5 s. SICI/ICF was calculated as the ratio of the mean conditioned MEP over the mean unconditioned test MEP. The unconditioned test MEPs in SICI/ICF assessments were used to evaluate any change in the size of MEP.

### 2.6. Experiment 3: The Effect of EcTBS on Spinal Reciprocal Inhibition

In a separate experiment, RI in the same forearm was assessed. RI assesses the interaction between stimulation of the radial nerve and the H-reflex produced by stimulation of the median nerve [33]. Electrical median and radial nerve stimulation were performed through surface electrodes. One electrical stimulator was used to stimulate the median nerve in the antecubital fossa. Stimulation duration lasted for 1 ms, and the intensity of the stimuli was adjusted to elicit approximately half-maximal H-reflexes from FCR in the baseline condition. The other electrical stimulator was used to stimulate the radial nerve in the spiral groove. The duration of the stimulus was 500 *μ*s, and the intensity was adjusted to produce an M wave around 50 *μ*V from EDC [34–36]. We recorded the H-reflex size during stimulation of the median nerve alone and for radial-medial stimuli at ISIs of −1, 0, 5, 10, 50, 100, and 300 ms, respectively. Stimuli were given every 8–12 sec in a random order for a total of ten trials per condition. RI was calculated as the ratio of the mean radial-median H-reflexes at each ISI over the mean unconditioned median alone H-reflex. RI was assessed before and at 30 min after EcTBS1200 at 90% H-reflex threshold. Two more subjects were recruited in this experiment because the initial result of phase 2 was unclear.

### 2.7. Experiment 4: The Effect of EcTBS on Post-Activation Depression

PAD was tested before and 30 min after spinal EcTBS1200 at 90% H-reflex threshold in the same forearm to understand the presynaptic mechanism of the spinal reflex in EcTBS. Electrical stimulation was performed via surface electrodes. Stimulation duration lasted for 1 ms, and the intensity of the stimuli was adjusted to evoke maximal H-reflexes from FCR in the baseline condition. Four conditions composed of two electrical median nerve stimuli at ISIs of 1, 3, 5, and 7 sec were given every 30 ± 10% sec in a random order for a total of six trials in each condition. PAD was calculated as the ratio of the conditioned (second) H-reflexes to the unconditioned (first) H-reflex at each trial and was averaged for each condition.

Experiments were done in different days at least two weeks apart from each other in a random order.

### 2.8. Data Analysis

A two-way repeated measure ANOVA on values normalized to the baseline with factors of TIME (0, 15, 30, 45, and 60 min) and PROTOCOL were applied to compare the effects on the H/M ratio between different EcTBS protocols. A one-way ANOVA analysis on nonnormalized values followed to test the effect of TIME (baseline, 0, 15, 30, 45, and 60 min) for the effect of the individual protocol. The results of MEP, SICI/ICF, and RI were analyzed by the paired *t*-test (baseline and 30 min after EcTBS1200). For PAD, a two-way ANOVA with factors of TIME (before and 30 min after) and ISI (1, 3, 5 and 7 sec) was performed to compare PAD before and after EcTBS. Furthermore, a one-way ANOVA on unconditioned baseline H-reflex and conditioned H-reflexes at ISI of 1, 3, 5, and 7 sec followed by post hoc tests was performed to test the effect of ISI on PAD. Differences were considered significant at a level of 5% or below.

## 3. Results

### 3.1. The Effect of EcTBS on H-Reflex

**Figure 1:**
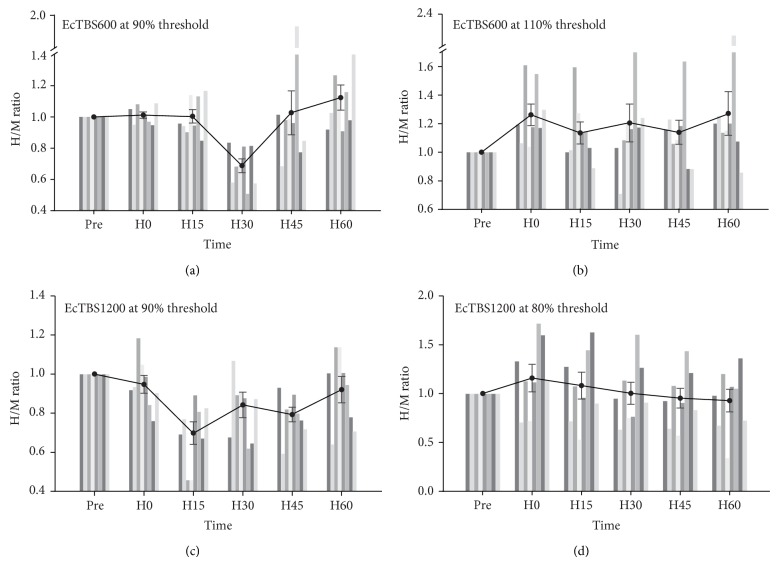
Different patterns of EcTBS lead to different effects on the H-reflex. Inhibition occurred, mainly at 30 min, after EcTBS600 at 90% threshold (a), while mild facilitation occurred after EcTBS600 at 110% threshold (b). In EcTBS1200, stimulation at 90% threshold inhibited the H/M ratio for more than 45 min (c), while EcTBS1200 at 80% threshold produced no after effect (d). The vertical bars are results of each subject.

There was no significant difference between the baseline H/M ratio in all conditions of experiment 1 (*F*(3,21) = 2.129, *P* = 0.127). The effect of EcTBS600 at different intensities was then compared on values normalized to the baseline (Figure 1(a)). A two-way repeated measure ANOVA for the H/M ratio revealed a significant interaction between INTENSITY (110% and 90% H-reflex threshold) and TIME (*F*(1,28) = 14.515, *P* = 0.007), for EcTBS600 at 110% H-reflex threshold facilitated the H/M ratio (one-way ANOVA: *F*(5,35) = 6.394, *P* < 0.001), while EcTBS600 at 90% H-reflex threshold inhibited the H/M ratio (one-way ANOVA: *F*(5,35) = 2.580, *P* = 0.043), mainly at 30 min after EcTBS. We then compared the effects of EcTBS1200 at 90% and 80% threshold and revealed an INTENSITY × TIME interaction (*F*(4,28) = 10.148, *P* = 0.015) (Figure 1(b)). A following one-way ANOVA confirmed that it was because of EcTBS1200 that at 90% threshold significantly suppressed the H/M ratio (*F*(5,35) = 3.173, *P* = 0.018), while EcTBS1200 at 80% threshold failed to produce any effect (*F*(5,35) = 1.953, *P* = 0.110). Furthermore, we compared the effects of EcTBS600 and EcTBS1200 at the same intensity of 90% threshold. Two-way ANOVA showed a significant interaction between TIME × DURATION (EcTBS600 and EcTBS1200) (*F*(4,28) = 3.187, *P* = 0.028), indicating that EcTBS1200 produced a more profound inhibitory effect than EcTBS600. We further tested the amplitude of Mmax and found that none of the protocols of EcTBS changed Mmax (one-way ANOVAs, all NS).

### 3.2. The Effect of EcTBS on Motor Cortical and Corticospinal Circuits

**Figure 2:**
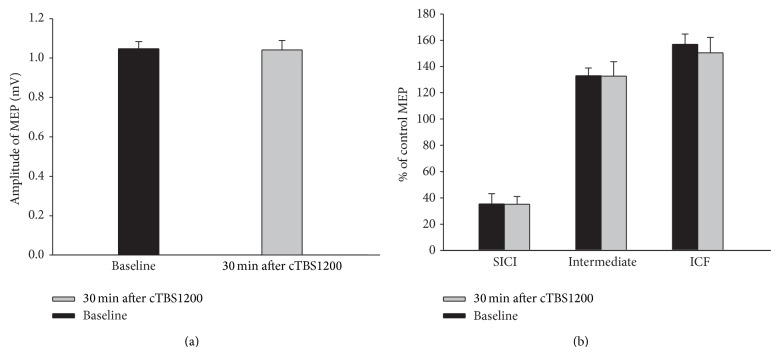
No changes on either TMS-induced MEP or SICI/ICF by EcTBS1200. There is no significant change on MEP (a) and SICI/ICF (b) after EcTBS1200.

Paired *t*-tests revealed that EcTBS1200 at 90% threshold did not alter the amplitudes of unconditioned MEP (*t* = 0.012; *P* = 0.917) (Figure 2(a)), SICI at ISI of 3 ms (*t* = 0.036, *P* = 0.972), ICF at ISI of 10 ms (*t* = 0.782, *P* = 0.460), and SICI/ICF at intermediate ISI (7 ms) (*t* = 0.032, *P* = 0.975) (Figure 2(b)).

### 3.3. The Effect of EcTBS on Spinal Reciprocal Inhibition

**Figure 3:**
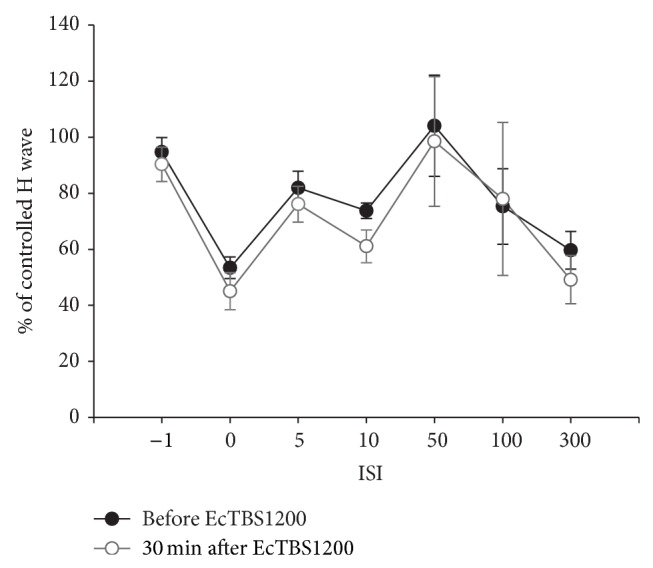
No change in RI before and after EcTBS. The amplitude of the H-reflex was inhibited by radial-medial stimulation at ISI of 0, 10, 100, and 300 ms. EcTBS1200 did not alter the curve of RI.

The results of RI (before and 30 min after EcTBS1200) at all ISIs are shown in Figure 3. Data were analyzed in three phases separately: phase 1 at ISI of 0 ms, phase 2 at ISI of 10 ms, and phase 3 at ISIs of 100 and 300 ms. We analyzed phase 3 by averaging the results at ISIs of 100 and 300 ms. Paired *t*-tests revealed no significant difference at all phases after EcTBS1200 (Phase 1: *t* = 1.455, *P* = 0.258; Phase 2: *t* = 3.597, *P* = 0.090; Phase 3: *t* = 0.071, *P* = 0.796).

### 3.4. The Effect of EcTBS on Postactivation Depression

**Figure 4:**
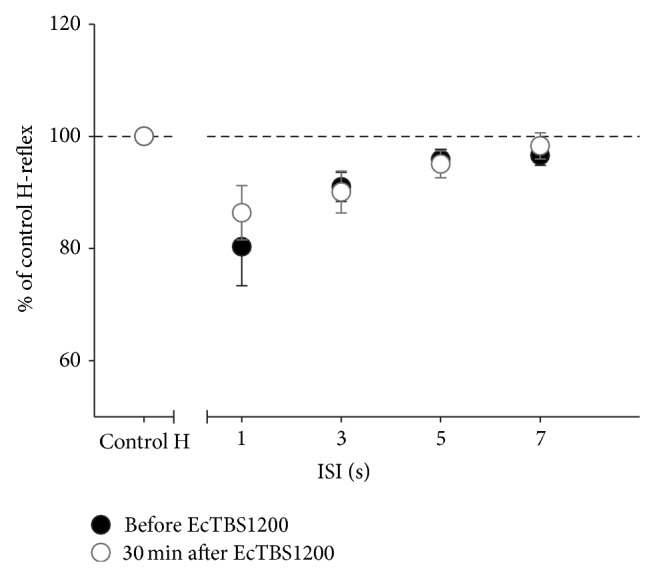
PAD was not changed by EcTBS1200. No significant difference was found in PAD before and after EcTBS1200. Additionally, the depression effect of PAD became nonsignificant after 5 seconds.

The results of PAD (before and 30 min after EcTBS1200) at all ISIs are shown in Figure 4. A two-way ANOVA revealed a significant effect of ISI (*F*(3,21) = 6.976, *P* = 0.002) but no significant effect of TIME (*F*(1,7) = 0.668, *P* = 0.441) and no TIME × ISI interaction (*F*(3,21) = 1.307, *P* = 0.298). The results indicate that PAD was not changed by EcTBS. We therefore put pre- and post-EcTBS data together to evaluate the effect of ISI on PAD. A one-way ANOVA showed a significant effect of ISI indicating that the second H-reflex was significantly suppressed by the first H-reflex. The post hoc test confirmed that the second H-reflex was significantly suppressed at ISI of 1, 3, and 5 second(s) but not at 7 seconds (*P* = 0.05, 0.06, 0.021, and 0.088, resp.).

## 4. Discussion

In our experiments, the H/M ratio was suppressed following EcTBS at 90% H-reflex threshold. Eighty seconds of EcTBS containing 1200 pulses suppressed the H/M ratio for 45 min or more, while 600 pulses of EcTBS produced a shorter-lasting inhibitory effect. Interestingly, increasing the stimulus intensity to 110% threshold did not enhance the suppressive effect but produced a facilitation instead. By contrast, 80% threshold produced no detectable effect on the H/M ratio. None of the protocols changed the size of Mmax. Moreover, spinal reciprocal inhibition, the size of MEP, and SICI/ICF remained unchanged at 30 min after EcTBS1200 at 90% threshold, when the H-reflex was most suppressed.

EcTBS modifies the H/M ratio and leaves the amplitude of Mmax unchanged, suggesting that EcTBS mainly affects the circuit of the H-reflex. The H-reflex involves a monosynaptic loop, in which the afferent consists of Ia fibers from the muscle spindles and the efferent consists of alpha motor axons, within the spinal cord [37]. The lack of change in Mmax after EcTBS indicates that EcTBS does not alter the excitability of the motor nerve, neuromuscular junction, and muscle. In addition, the TMS study showed no effect of EcTBS on the amplitude of MEPs and SICI/ICF, suggesting no involvement of excitatory and inhibitory circuits within the motor cortex and the corticospinal tract. Therefore the effect of EcTBS is more likely located at the stimulated level of the spinal cord. On the other hand, the effect of EcTBS that lasts for 45 min or more after subthreshold stimulation is apparently different from postactivation depression of the H-reflex that only lasts for seconds and requires suprathreshold stimulation [38]. It could be argued that we measured the H-reflex every 6–12 sec in experiments 1 and 3 and the influence of the postactivation depression should be considered at this ISI. The results of the PAD test in the present study suggest that the depression effect becomes nonsignificant after 5 seconds. This is in line with most previous studies which showed that significant postactivation depression caused by the previous stimulus lasted no longer than 6–8 s [38–40]. It was also mentioned that H-reflexes were large and stable when they were recorded every 6 sec. Therefore, we believe that the possibility of an influence of postactivation depression is low in the present study.

To further clarify the mechanism of EcTBS at the spinal level we examined spinal reciprocal inhibition in the same nerve. There are three known phases of RI in the forearm. The first phase that occurs at ISI of 0 ms or so in the radial-medial stimulation is regulated by the glycinergic disynaptic inhibitory pathway, while the second phase that occurs at ISI of 10–20 ms includes the presynaptic Ia inhibitory pathway [33, 41]. The mechanism of the third phase that occurs at ISI of 70- several hundred ms is less clear and might be due to the polysynaptic stretch reflex pathway [42]. In the current study, all phases of RI remained unchanged after EcTBS, indicating that the effects of EcTBS were limited to the H-reflex circuit and that the effect is specific to the conditioned pathway. This result is in contrast to the enhanced reciprocal Ia inhibition on the H-reflex induced by patterned sensory peroneal nerve stimulation for 30 min [19]. Such differences between the effects of the two patterned stimulations may be due to differences in the stimulation protocol as well as its duration and slightly different stimulus intensity.

Moreover, we tested PAD to identify the location of the plasticity within the synapse of the H-reflex. Postactivation depression was thought to be due to the presynaptic mechanism involving a decreased probability of neurotransmitter release from previously active Ia-afferent terminal [43–46]. No change in PAD after EcTBS suggests that the presynaptic terminal of the H-reflex synapse is not modified by EcTBS. Together with no change in Mmax, this suggests that the effect of EcTBS is likely due to changes in the transmission efficiency of the monosynaptic connection within the H-reflex through mechanisms of postsynaptic plasticity that are commonly induced by repetitive presynaptic stimulation [47].

At first glance, we were surprised to see that EcTBS600 at 110% threshold facilitated the H-reflex. cTBS600 using rTMS generally shows an inhibitory effect over the cerebral cortex [30], although a recent report suggests that the effect of cTBS may be variable [48] and a shorter form of cTBS (i.e., cTBS for 20 sec) may produce slightly facilitation when there is no muscle activity before the stimulation [49]. Recently, the effect of TBS over the motor cortex was shown to be sensitive to the stimulus intensity [50]. Thus, the difference in intensity could explain the opposite effects of EcTBS at 90% and 110%. Although the exact reason for this is unclear, it might be explained by the theoretical model of TBS [51]. In this model, repetitive stimulation was hypothesized to induce a mixed effect of facilitation and inhibition. The effect on the synapse depends on the summation of potentiation and depression. It is possible that a higher stimulus intensity favors potentiation effects since a higher intensity may cause a larger amount of Ca^2+^ influx that tends to induce LTP [52, 53]. Alternatively, it is also possible that suprathreshold stimulation activates pre- and postsynaptic terminals simultaneously to produce an effect different from those induced by presynaptic subthreshold stimulation. In contrast, stimulus intensity below a certain level, for example, 80% threshold, may not activate the circuit effectively and thus produces no after effect.

Different from EcTBS over M1, EcTBS600 at 90% threshold only produced a relatively short-lasting inhibitory effect around 30 min after the end of EcTBS, indicating that spinal circuits are less modifiable than those in the brain. It is reasonable since the brain needs to be more flexible than the spinal cord for, say, learning and memorizing. After doubling the length of stimulation, EcTBS1200 is capable of producing a more profound and consistent inhibitory effect for 45 min or more. This result implies that, at least within a certain range, the longer the stimulation lasts, the stronger and longer-lasting after-effect on the H-reflex it may produce. This is consistent with a previous report of TBS using rTMS [54]. Nevertheless, not all studies agree with this hypothesis [55]. The complicated mechanism of TMS may contribute to such inconsistent results of the TBS form of rTMS. Electrical activation of the median nerve to elicit H-reflexes only involves a loop with a single synapse and their underlying mechanisms are simpler and clearer when compared to that of TMS. Hence, we expect that the study of spinal plasticity using the present protocol provides another approach for studying synaptic plasticity in conscious human beings.

## 5. Conclusion

EcTBS modulates the H-reflex likely through a mechanism involving synaptic plasticity in the human spinal cord. The effect of EcTBS depends on its stimulus intensity. Intensities higher than the H-reflex threshold tend to facilitate the H-reflex, while intensities slightly below the threshold suppress H-reflexes. Moreover, a longer-lasting EcTBS induces a more consistent and longer-lasting after-effect than a shorter EcTBS. Hence, EcTBS1200 at 90% threshold is an efficient protocol to produce an inhibitory LTD-like effect in the human spinal cord and it can be used for exploring mechanisms of human plasticity, understanding the pathophysiology of diseases relevant to spinal plasticity and, hopefully, providing therapeutic benefits on spinal disorders in the future.
